# Novel Techniques and Technologies for Surgical Aortic Valve Replacement: A Large Retrospective Cohort Analysis

**DOI:** 10.3390/jcm13144126

**Published:** 2024-07-15

**Authors:** Vincenzo Caruso, Rajdeep Bilkhu, Christopher Young, James Roxburgh, Paolo Bosco, Gianluca Lucchese

**Affiliations:** Cardiovascular Department, St. Thomas’ Hospital, London SE1 7EH, UK; rajdeep.bilkhu@gstt.nhs.uk (R.B.); christopher.young@gstt.nhs.uk (C.Y.); james.roxburgh@gstt.nhs.uk (J.R.); paolo.bosco@gstt.nhs.uk (P.B.); gianluca.lucchese@gstt.nhs.uk (G.L.)

**Keywords:** aortic valve replacement, trans-aortic valve implantation, minimally invasive surgery, ross operation, sutureless aortic valves

## Abstract

**Background/Objectives**: In an era of growing evidence for transaortic valve implantation (TAVI), our research topic was the evaluation of how surgical aortic valve replacements (SAVRs) are performing in terms of short- and long-term outcomes in different risk categories. **Methods**: This was a single centre, prospective, and observational cohort study of consecutive patients with aortic valve stenosis, undergoing isolated aortic valve replacement using a biological or mechanical prosthesis, Freestyle™ (Medtronic, Minneapolis, MN, USA) graft, homograft, or Ross procedure. The participant data were collected by review of an internal database. The primary endpoints were all-cause operative mortality (in hospital and at 30 days) and late mortality at the follow-up date. The secondary composite endpoint was the incidence of postoperative complications. **Results**: 1501 patients underwent SAVR; the mean age was 67 years (SD: 12.6). The in-hospital mortality was 1% (*n* = 16). At a median follow-up of 60 months, the survival rate was 98.7%. The main predictors for mortality were operative urgency and cardiogenic shock. The overall incidence of PPM was 2.3% (*n* = 34). Patients who underwent Ross procedure were younger (mean age: 20 years (SD: 1.7)), had a lower incidence of postoperative complications, and were all alive at follow-up. **Conclusions**: SAVR shows an excellent survival rate and a low rate of postoperative complications despite an increasing surgical risk. Recent advancements in technology, like sutureless/rapid deployment prostheses and minimally invasive techniques, are shown to have favourable effects on outcomes.

## 1. Introduction

Surgical aortic valve replacement (SAVR) is still considered the gold standard treatment for aortic valve replacement (AVR), with transcatheter aortic valve implantation (TAVI) reserved for those patients with a prohibitive or higher surgical risk [1]. TAVI has evolved over the years, being utilised now in lower-risk and younger patients [2,3,4,5]. American guidelines have introduced TAVI as the preferred technique for valve replacement in patients >80 years old or for younger patients with a life expectancy <10 years [6]. European guidelines recommend TAVI for patients >75 years old or in those who are high risk or unsuitable for surgery [7]. Growing evidence has endorsed TAVI as a viable alternative to SAVR [8,9,10].

Conversely, SAVR has undergone refinement of its technique with implementation of the use of sutureless valves [11,12] and minimally invasive approaches [13,14,15].

Patients considered for SAVR now have increased comorbidity [16]; most series of SAVR in the literature tend to report the overall outcomes [17,18,19], but they do not stratify based on surgical risks and indications for surgery.

The aim of this study is to evaluate the clinical outcomes of patients undergoing isolated SAVR at a single centre. The study seeks to stratify these outcomes based on the following several key factors: preoperative patient characteristics, different surgical techniques employed, and types of prosthetic valves used. By analysing these variables, the study intends to uncover patterns and correlations that can inform better preoperative planning and intraoperative decision making.

## 2. Materials and Methods

### 2.1. Study Design and Endpoints Definition

This was a retrospective analysis of prospectively collected data from a single institution. Data for all adult patients undergoing isolated SAVR, regardless of operative urgency, from April 2011 to December 2019, were collected from the departmental database. Patients undergoing transapical TAVI, concomitant cardiac surgery, and/or major aortic surgery were excluded from the analysis. The patients were followed until May 2020.

Data on the preoperative demographic characteristics, echocardiographic variables, intra-operative data, type of aortic valve prosthesis (biological, mechanical, homograft, or Ross procedure), and postoperative complications were collected. The surgical approach for each case was also collected, i.e., full median sternotomy (FMS), mini-sternotomy (M-S), and right anterior thoracotomy (RAT). The operative urgency was also recorded, with elective being defined as when the patient was admitted from home; urgent meaning the patient required surgery during the same hospital admission; and emergency and salvage meaning that surgery was required within 24 h of admission and/or the patient was in extremis.

The primary endpoints were all-cause operative mortality, defined as in-hospital mortality or 30-day mortality, including patients who died after 30 days but without discharge, and all-cause late mortality at the follow-up date. Operative mortality was also evaluated in relation to preoperative characteristics, echocardiographic variables, and intra-operative data.

The secondary composite endpoint was the incidence of postoperative complications. These were transient ischemic attack (TIA), defined as a transient (less than 24 h) episode of neurologic dysfunction, or stroke, defined as persistent (more than 24 h) neurological deficits and confirmation of brain infarction or haemorrhage on imaging; permanent pacemaker implant (PPM), defined as implant of an external pacemaker within 30 days from surgery; acute kidney failure (AKI), defined as a decline in renal function with need for dialysis; immediate (defined as less than 24 h from surgery) return to the theatre for postoperative bleeding and/or pericardial effusion or cardiac arrest; early valve dysfunction, defined as aortic valve failure within 30 days from surgery; and early superficial or deep sternal wound infection (DSWI), defined as wound infection occurring within 30 days from surgery.

An additional analysis was performed to evaluate the different composite outcomes for the surgical approaches or types of prostheses used.

### 2.2. Statistical Analysis

Statistical analysis was conducted using the STATA version 18 software package and the R studio software package, version 2024.04.02+765.

Continuous data are expressed as means and standard deviations (SDs) or medians and interquartile ranges (IQRa); categorical variables are reported as counts and percentages.

The normality of distribution of the continuous variables was tested by the Shapiro–Wilk test. Data analysis bias was reduced with the following statistical corrections: logarithmic transformation of skewed data was used for logistic regression; missing values (missingness identified as completely at random—MCAR) were replaced using both linear interpolation and multiple imputation; and outliers were transformed with logarithmic function. Cumulative probability for death was estimated by use of the Kaplan–Meier method, and log-rank was employed to detect differences among curves.

Univariate analysis was performed using the Pearson’s chi-square test or the Fisher exact test. Significant variables were then included in a multivariate Cox-regression analysis, which was employed to detect predictors of late death.

Differences in postoperative outcomes were detected using bivariate correlation analysis and the exact chi-square test. The outcomes of the different prostheses implanted were assessed with the *t*-Test, Kruskal–Wallis test, or Mann–Whitney U test. Binary and multivariable regressions were used to identify potential predictors for incidence of postoperative complications. For all tests, a *p*-value < 0.05 was considered statistically significant.

## 3. Results

Between April 2011 and December 2019, 1501 patients underwent SAVR in a single unit. The mean age was 67.5 years (SD: 12.6), and 202 (13.5%) were octogenarians (Table 1).

Aortic stenosis was the most common indication for SAVR (*n* = 1142, 76.1%) with 1129 cases (75.2%) due to calcific degeneration; active or subacute infective endocarditis (IE) was instead an indication for AVR in 85 patients (5.7%).

Emergency or salvage surgery was performed in 33 patients (2.2%); twenty-two (1.5%) presented with cardiogenic shock requiring inotropic support, intubation prior to surgery (*n* = 20, 1.2%), and intra-aortic balloon pump (*n* = 5, 0.3%) (Table 2).

### 3.1. Mortality

#### 3.1.1. All-Cause Operative Mortality

The all-cause operative mortality, as previously defined, was 1% (*n* = 16). The mean age of these patients was 71.5 years (SD: 10.8) and death occurred at a median of 6.5 days (IQR: 4, 15). Seven of these patients underwent nonelective surgery (44%); three (18.7%) were in cardiogenic shock (Table 3).

#### 3.1.2. All-Cause Late Mortality

**Figure 1:**
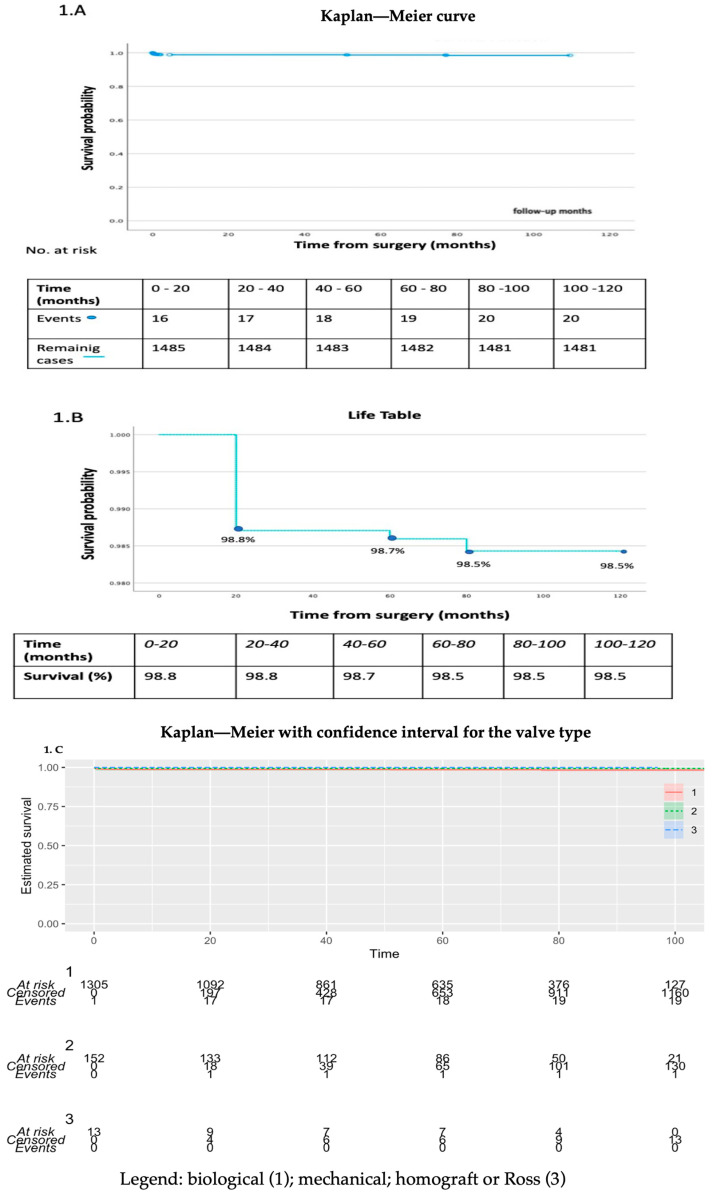
Mortality and postoperative complications: (**A**) Kaplan—Meier for overall cohort; (**B**) Life table for overall cohort; (**C**) Kaplan—Meier with confidence interval for valve type (KMunicate package—R Studio).

At a median follow-up of 60 months (IQR: 31, 85), survival was 98.5%, with a mortality of 1.3% (*n* = 20) (Figure 1A–C).

The univariable analysis demonstrated that preoperative variables associated with mortality were the New York Heart Association class, preoperative renal dialysis, pulmonary disease, ejection fraction, timing of surgery, and postoperative renal and neurological dysfunctions.

The multivariable analysis demonstrated that cardiogenic shock (HR: 25 (9, 69), *p* = 0.017), operative urgency (HR: 0.3 (0.1, 0.7), *p* = 0.007), postoperative renal failure (HR: 100 (40, 246), *p* = 0.001), and neurological dysfunction (HR: 4.5 (2.6, 8), *p* = 0.001) were the only variables associated with higher mortality.

The age-related survival analysis demonstrated no significant differences among the age groups, despite the rate of mortality being lower in the group of patients younger than 50 years old (mean survival years—18–50 years old: 9 (SD: 0.7) (95% CI: 8.9, 9.1); 50–70 years old: 9 (SD: 0.4) (95% CI: 8.9, 9.08); 70–79 years old: 8.9 (SD: 0.5) (95% CI: 8.8, 9); and >80 years old: 9 (SD: 0.4) (95% CI: 8.9, 9), *p* = 0.869).

The survival analysis demonstrated that patients operated on as urgent or emergency experienced a significantly higher mortality than those operated on as elective cases (mean survival months: 106.3 (SD: 0.9) versus 108.6 (SD: 0.3), respectively, *p* = 0.001).

The aetiology of valvular disease did not influence mortality (log-rank test: *p* = 0.781).

No differences were noted in terms of mortality based on surgical approach (FS: *n* = 13, 1.4%; M-S: *n* = 6, 1.3%; RAT: *n* = 1, 0.8%; log-rank test: *p* = 0.861).

Increased mortality rates were noted with the Freestyle™ (Medtronic, Minneapolis, USA) bioprosthesis in contrast to both biological and mechanical valves, whereas comparable outcomes were observed between the homograft or Ross procedure and biological and mechanical valves (biological valve, *n* = 16, 1.3%; mechanical, *n* = 2, 0.8; Freestyle™, *n* = 2, 11.1%; homograft, *n* = 0; Ross, *n* = 0; pairwise Biological/Freestyle™: *p* = 0.002; pairwise mechanical/Freestyle™: *p* = 0.003).

### 3.2. Postoperative Outcomes

#### 3.2.1. Postoperative Neurological Events

The incidence of stroke was 0.4% (*n* = 6), and TIA occurred in 19 patients (1.3%). Patients with a calcified aortic valve had a higher incidence of neurological events, but this was not statistically significant (*n* = 21, 1.4%, odds ratio (OR): 1.5 (0.5, 4.6), *p* = 0.481). Gender or age were not predictive for neurological dysfunction (*p* = 0.74 and *p* = 0.212, respectively).

#### 3.2.2. PPM Implant

The overall incidence of PPM was 2.3% (*n* = 34). The cohort characteristics are shown in Table 4. Sutureless aortic valves had an incidence of 2% (*n* = 3), with no significant differences between sutured and sutureless or rapid deployment aortic valves (*p* = 0.621).

#### 3.2.3. Postoperative Renal Failure

Twenty-one (1.4%) patients required dialysis for acute kidney injury. Preoperative renal failure *(n* = 34, 2.4%) was significantly associated with incidence of postoperative dialysis (*n* = 6, 29%, OR: 16.5 (5.9, 44.7), *p* < 0.001).

#### 3.2.4. Return in Theatre

Re-exploration for bleeding was performed in 32 patients (2.1%). Re-exploration for cardiac arrest or haemodynamic instability was performed in 6 patients (0.4%).

#### 3.2.5. Sternal Wound Infection

The incidence of DSWI was 1.3% (*n* = 20). Of these, one (0.1%) required return to theatre for wound debridement and sternal rewiring.

### 3.3. Comparison of Prostheses

Biological valves (*n* = 1197, 79.7%) were implanted more often than mechanical ones (*n* = 243, 16.2%).

Other devices were Freestyle™ aortic or aortic root valves (*n* = 18, 1.2%) and homograft (*n* = 10, 0.7%). The Ross procedure was performed on 33 patients (2.2%) (Table 5).

The patients for which a homograft was used or who underwent the Ross procedure, were younger (mean age: 51.3 years (SD: 18.9) and 20 years (SD: 1.7), respectively) and were all alive at follow-up. In these groups, the incidence of postoperative complications was lower; this did not reach statistical significance, except for postoperative dialysis.

#### Biological versus Mechanical Valves

Between the two groups, there was a significant difference in age as expected (mean years: 69.3 (SD: 10.6) and 60.3 (SD: 14.2, *p* < 0.001)) and operative times (CPB minutes: 77 (SD: 26.4) and 84.4 (SD: 31.4), *p* = 0.001; XCT minutes: 60 (SD: 20.1) and 65.3 (SD: 18.8), *p* = 0.001).

Despite this, mortality was similar between the two groups (mean survival days at follow-up: biological valve, 108.42 (SD: 11); mechanical valve, 108.85 (SD: 19), *p* = 0.513).

**Figure 2:**
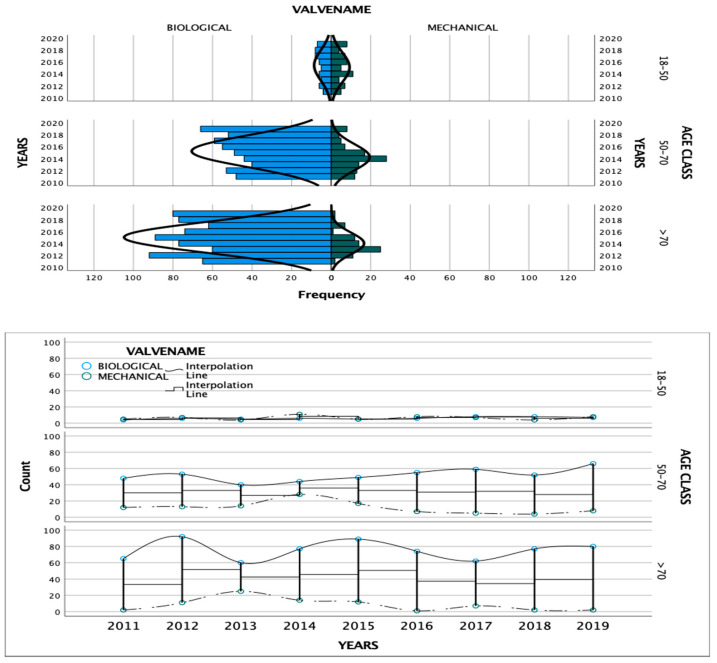
Comparison of biological and mechanical valves.

Biological valves were often used in those patients 50–70 years old and >70 years. In the 18–50 years old age group, a similar number of patients received either a mechanical or bioprosthetic valve; there was an increase in the use of biological valves for the 50–70 years age group over time (Figure 2), and this was statistically significant from 2016 to 2019 (2016: rho= 0.265, *p* = 0.01; 2018: rho = 0.241, *p* = 0.05; 2019: rho = 0.322, *p* = 0.01).

### 3.4. Comparison of Surgical Approaches

With the exclusion of the Freestyle™, homograft implant, and Ross procedure, the M-S approach had shorter surgical times compared with the FMS (CPB minutes: 71.4 (SD: 12.2) versus 65.4 (SD: 19.4); XCT minutes: 56.8 (SD: 10.5) versus 51.3 (SD: 16.3)), but this was not significantly different.

The RAT approach had significantly longer surgical times compared with FMS and M-S (CPB minutes: 10 (SD: 21.5); XCT minutes: 78.7 (SD: 16), *p* < 0.001). However, no significant differences were noted regarding mortality (χ^2^ = 0.886), DSWI (χ^2^ = 0.703), AKI (χ^2^ = 0.890), or return to theatre for any causes (χ^2^ = 0.742), while statistical significance was noted in the incidence of postoperative neurological events (χ^2^ < 0.001, FS: *n* = 9, 1.1%; M-S: *n* = 8, 1.8%; RAT: *n* = 7, 5.5%).

## 4. Discussion

In this study, we report a detailed contemporary analysis of the outcomes and long-term survival following isolated SAVR. We report excellent long-term survival with a mortality of 1.3% at the follow-up date (median: 5 years (IQR: 2.5–7)). This is in keeping with recent studies, showing a higher mortality for those patients managed medically [20,21].

From our results, calcified aortic stenosis remains the common indication for SAVR, with bicuspid aortic valve (BAV) becoming increasingly common [22].

Also the incidence of IE has increased [23,24]; in our cohort, we report an increased incidence of IE over the years (2013: 3.8%; 2019: 8.6%).

From our data, it was found that age was not an independent risk for mortality and there was no difference in term of mean survival time across the different age groups. Therefore, age alone may not be considered a factor in the decision for surgery versus TAVI or medical treatment.

Cardiogenic shock pre-surgery remains an important risk factor, and it was associated with a poor prognosis. It can, therefore, be suggested that the most important determinant of a worse outcome is the urgency of the surgery itself, independently of other associated risk factors. This is an important point when considering the outcomes of TAVI. Despite the growing evidence for TAVI as an alternative to SAVR [8,9,10], the outcomes for patients undergoing TAVI as an urgent or salvage procedure remain unclear and limited to case reports [25,26]. Recently, the OCEAN-TAVI [27] registry demonstrated that the urgency, itself, was not a predictor for mortality for TAVI, but the cohort of patients undergoing nonelective TAVI, in this study, was limited only to 5.4%. In our current study, which reports mortality in all categories of operative urgency, 33% of patients underwent a nonelective operation.

Even considering the increasing risk profile of patients undergoing operation over time (due to age, IE, comorbidity, or prolonged life expectations), the trend in mortality, in our study, showed a decreasing incidence across the years. This is consistent with findings from a recent study, from Japan, which assessed outcomes of SAVR from their national database and in which they demonstrated reduced mortality over time despite increasing surgical risk [15]. This may be due to the implementation of the use of new technologies, e.g., sutureless valves [11,12] and minimally invasive techniques [13,14]. These findings should be considered when comparing the SAVR to the TAVI, especially in those patients with similar risk factors.

Of note, in the current study, neurological events appeared to have greater incidences in elective patients. Possible reasons may be the aetiology of aortic valve disease, as with a calcified aortic valve, the primary indication for SAVR in elective patients is associated with an increased risk of postoperative stroke. Furthermore, the incidences of neurological events were greater in patients undergoing RAT. It is important to note though that these cases represent our initial experience with RAT, and the increased TIA/stroke incidence might be related to the longer XCT and CPB times because of our initial learning curve. In the entire cohort, the occurrence of PPM stood at 2.3% (*n* = 34), with no significant difference in the PPM frequency observed between sutured and sutureless/rapid deployment prostheses. Interestingly, sutureless/rapid deployment valves have been linked to a heightened risk of PPM implantation [28]; however, various technical adaptations and insights have emerged in recent years [29,30]. Our findings suggest that the low incidence of PPM in our cohort can be attributed to improved intraoperative sizing techniques and the growing experience of performing surgeons.

The current study demonstrated a change in the trend of the type of prosthetic valve implanted. In recent years, there has been an increase in bioprosthetic valves implanted in patients younger than 65 years old at our centre. None of the patients in the current study required a redo of surgery for structural valve deterioration; however, the follow-up in this study was limited to 5 ± 2.1 years.

Our experience with the Ross procedure, which was performed in a subcoronary fashion, is consistent with very good outcomes in terms of mortality and freedom from autograft and/or homograft reinterventions. However, of note, when compared to the results of biological and mechanical valves, no significant differences were observed in terms of mortality. With a rejuvenated interest in the Ross procedure [31,32], the ability to perform it in a tertiary cardiac centre is a valid addition to the surgical armamentarium in treating aortic valve disease, particularly in younger patients referred for SAVR, many of whom do not wish to be on lifelong anticoagulation. However, the Ross procedure remains a highly specialised procedure, and our centre is unique in its ability to offer this technique.

In term of minimal access surgery, our results demonstrate shorter surgical times for M-S when compared with FMS; this may be due to different reasons. First, almost 40% of the SAVRs on FMS in our study were performed by trainees; second, M-S is accompanied by the use of sutureless valves, if appropriate, and this might justify a shorter implantation time [11,12]. The RAT approach still showed longer cardiopulmonary and cross-clamp times; despite this, length of stay and mortality were similar to other surgical approaches.

The findings of this study highlight the need for future research, moving the surgical attitude to the development of new prosthetic valves and minimally invasive approaches and refinement of procedures such as Ross; furthermore, the variability in outcomes across different surgical procedures and patient populations suggests that more targeted research is needed to identify specific factors that contribute to successful surgical implantation.

The strengths of the study include the fact that all patients of all surgical risks were included. The limitations include its retrospective nature and the fact that long-term functional assessment of patients, neither diagnostic nor hemodynamic studies, were performed to assess the function of the implanted valves. This was due to geographical reasons and the fact that not all patients were followed up at our unit.

## 5. Conclusions

Our results show that SAVR has excellent survival and a low rate of postoperative complications, despite increasing surgical risk. Minimally invasive approaches do not impact mortality and show satisfactory outcomes. In addition, prosthetic AVR has a comparable long-term survival to the Ross operation in our experience. Our data support SAVR as a first-line approach in the management of patients with aortic valve disease.

## Figures and Tables

**Table 1:** Preoperative demographic characteristics, risk factors, and echocardiographic variables.

	*n* = 1501	
Age at Surgery (years)	Mean and (SD)	67 (SD: 12.6)
Octogenarian	*n* (%)	202 (13.5)
Gender	*n* (%)	
Male		931 (62)
Female		570 (38)
EuroScore (ES)	Median (IQR)	
Logistic ES		4.25 (2.4, 7)
ES 2 or II		2.8 (1–2, 6)
NYHA	*n* (%)	
I		146 (9.7)
II		461 (30.7)
III		735 (49)
IV		159 (10.6)
Hypertension	*n* (%)	945 (63)
Diabetes	*n* (%)	
No		1240 (82.6)
Type 1		50 (3.3)
Type 2		211 (14.1)
Smoking	*n* (%)	
Never		670 (44.6)
Ex-smoker		665 (44.3)
Smoker		166 (11.1)
Renal Function	*n* (%)	
Preserved		1460 (97.3)
Dialysis		20 (1.3)
Chronic kidney disease		5 (0.3)
Acute kidney disease		16 (1.1)
Creatinine	mmol/l	90 (SD: 40.5)
Neurological Disease	*n* (%)	
No		1399 (93.2)
TIA		72 (4.8)
Stroke		17 (1.1)
Rhythm	*n* (%)	
Sinus rhythm		1344 (89.5)
Atrial fibrillation		110 (7.3)
Complete heart block		22 (1.5)
Ventricular fibrillation		1 (0.1)
Other *		11 (0.7)
Ejection Fraction (%)	Mean (SD)	54 (SD: 9.8)
Good		1166 (77.7)
Moderate		242 (16.1)
Poor		58 (3.9)
Very poor		4 (0.3)
Cardiogenic Shock	*n* (%)	22 (1.5)
Inotropes	*n* (%)	22 (1.5)
Ventilation	*n* (%)	20 (1.2)
IABP	*n* (%)	5 (0.3)
Timing	*n* (%)	
Elective		1156 (77)
Urgent		312 (20.8)
Emergency		29 (1.9)
Salvage		4 (0.3)
Coronary Disease	*n* (%)	
No coronary disease		1330 (88.6)
1—vessel disease		95 (6.6)
2—vessel disease		76 (5)
Aortic Valve Pathology	*n* (%)	
Stenosis		1142 (76.1)
Regurgitation		203 (13.5)
Mixed		156 (10.4)
Aetiology	*n* (%)	
Congenital		179 (11.9)
Degenerative		1129 (75.2)
Infective endocarditis		85 (5.7)
Functional disease		36 (2.4)
Other *		72 (4.8)
Effective Orifice Area Indexed (cm^2^)	Mean (SD)	0.7 (SD: 0.17)
Mean Gradient (mmHg)	Mean (SD)	80.9 (SD: 22)

SD: standard deviation; IQR: interquartile range; NYHA: New York Heart Association class; TIA: transient ischemic attack; IABP: intra-aortic balloon pump; CPB: cardiopulmonary bypass; XCT: cross-clamp. * Rheumatic disease, traumatic rupture, rescue after TAVI failure, aortic masses, and inflammatory disorders.

**Table 2:** Intra-operative data.

Intra-Operative Data		*n* = 1501
Primary Incision	*n* (%)	
Sternotomy		905 (60.3)
Upper hemi sternotomy		469 (31.2)
Right anterior thoracotomy		127 (8.5)
Aortic Valve	*n* (%)	
Tissue valve		1191 (79.7)
Mechanical valve		243 (16.2)
Freestyle		18 (1.2)
Homograft		10 (0.8)
Ross autograft		33 (2)
CPB (minutes)	Mean (SD)	82.3 (SD: 35.2)
XCT (minutes)	Mean (SD)	64.4 (SD: 27)
Length of Stay (days)	Median (IQR)	7 (6, 10)
Follow-Up (months)	Median (IQR)	60 (31, 85)

CPB: cardiopulmonary bypass; XCT: cross-clamp.

**Table 3:** Mortality and postoperative complications.

Postoperative Data		n = 1501
Operative Mortality *	*n* (%)	16 (1)
Overall Late Mortality **		20 (1.3)
Elective		10 (0.9)
Urgent		6 (1.9)
Emergency		3 (10.3)
Salvage		1 (25)
Permanent Pacemaker Implant	*n* (%)	34 (2.3)
Neurological Events	*n* (%)	
Transient ischemic attack		19 (1.3)
Stroke		6 (0.4)
Acute Kidney Injury/Dialysis	*n* (%)	21 (1.4)
Re-Exploration	*n* (%)	40 (2.7)

* All-cause in-hospital mortality or 30-day mortality including patients who died after 30 days but without discharge. ** All-cause mortality at the follow-up date.

**Table 4:** Characteristics of PPM’s cohort.

Cohort		*n* = 34
Mean Age	years	69.9 ± 11.6
Sex	*n* (%)	
Male		19 (56)
Female		15 (44)
Preoperative Rhythm	*n* (%)	
Sinus rhythm		27 (79.4)
Atrial fibrillation		7 (20.6)
Beta-Blocker Use	*n* (%)	13 (38.2)
Bicuspid Valve	*n* (%)	6 (17.6)
Valve Types	*n* (%)	
Perimount 2900		17 (50)
Magna Ease		8 (23.5)
Intuity		3 (8.8)
On-X		2 (5.9)
Trifecta		1 (2.9)
St. Jude		1 (2.9)
Inspiris		1 (2.9)

**Table 5:** Characteristics of the cohort according to valvular implant.

Variable	Biological	Mechanical	Freestyle	Homograft	Ross	*p*-Value
	(*n* = 1197)	(*n* = 243)	(*n* = 18)	(*n* = 10)	(*n* = 33)	
	−79.70%	−16.20%	−1.20%	−0.70%	−2.20%	
Age, years (mean ± SD)	69.2 ± 10.6	60.3 ± 14.2	61.6 ± 11.9	51.3 ± 18.9	20 ± 1.7	0.001 **
Gender, n (%)						0.080 *
Male	743 (81.8)	145 (16)	15 (1.7)	3 (0.3)	22 (1.5)	
Female	454 (80.6)	98 (17.4)	3 (0.5)	7 (1.2)	8 (0.5)	
Ejection Fraction, % (mean ± SD)	53.9 ± 9.5	53.2 ± 10.9	53.2 ± 13.8	58.5 ± 5.2	65 ± 0.1	0.789
Aetiology, n (%)						0.001 **
BAV	126 (10.5)	26 (10.7)	0 (0)	0 (0)	30 (91)	
Calcific	931 (77.8)	184 (75.7)	1 (5.6)	6 (60)	0 (0)	
IE	65 (5.4)	13 (5.3)	5 (27.8)	3 (30)	3 (9)	
Functional	25 (2.1)	4 (1.6)	6 (33.3)	1 (10)	0 (0)	
Other	50 (4.2)	16 (6.6)	6 (33.3)	0 (0)	0 (0)	
Timing of Surgery, n (%tot)						0.001 **
Elective	927 (63)	186 (12.6)	10 (0.7)	3 (0.2)	30 (2)	
Urgent	248 (16.9)	55 (3.7)	2 (0.1)	4 (0.3)	3 (0.2)	
Emergency	20 (1.4)	0 (0)	6 (0.4)	3 (0.2)	0 (0)	
Salvage	2 (0.1)	2 (0.1)	0 (0)	0 (0)	0 (0)	
Mean Gradient, mmHG (mean ± SD)	81.2 ± 16.1	80.8 ± 16.3	80.2 ± 4	80.1 ± 11.5	81.1 ± 0.1	0.899
CPB Minutes (mean ± SD)	77.04 ± 26.4	84.4 ± 31.4	185.7 ± 86.3	152.6 ± 59.7	225.3 ± 94	0.001 **
XCT, minutes (mean ± SD)	60.08 ± 20.1	65.3 ± 18.8	138.5 ± 49.3	122.7 ± 43.4	162.3 ± 71.2	0.001 **
Mortality, n (%)	16 (80)	2 (10)	2 (10)	0 (0)	0 (0)	0.471
Postoperative AKI, n (%)	15 (1.3)	3 (1.3)	2 (11.1)	1 (10)	0 (0)	0.021 *
Postoperative Stroke/TIA, n (%)	22 (1.9)	2 (0.8)	1 (5.6)	0 (0)	0 (0)	0.808
Wound Infection, n (%)	1 (0.1)	0(0)	0(0)	0(0)	0(0)	0.994
LOS Days, median (IQR)	7 (5–12)	7 (6–14)	7.5 (4–14)	14 (8–20)	6 (1–8)	0.088

SD: standard deviation; BAV: Bicuspid aortic valve; IE: infective endocarditis; CPB: cardiopulmonary by-pass; XCT: cross clamp; AKI: acute kidney injury; TIA: transient ischemic attack; LOS: length of stay; IQR: interquartile range. * *p* < 0.05. ** *p* < 0.005.

## Data Availability

The data underlying this article will be shared upon reasonable request to the corresponding author.
